# Evaluating poultry by-product meal, tomato pomace, and azolla with or without Natuzyme supplementation as sustainable alternatives to fish meal for Nile tilapia (*Oreochromis niloticus*)

**DOI:** 10.1007/s11250-026-05001-0

**Published:** 2026-04-17

**Authors:** Ahmed M. Kamel, Manal M. Mahmoud, Mohammed T. Ibrahim, Heba A. Alian

**Affiliations:** Faculty of Veterinary Medicine, Department of Nutrition and Clinical Nutrition, Suez Canal University, Ismailia, 41522 Egypt

**Keywords:** Azolla, Fish meal, Growth, Natuzyme, Poultry by-products meal, Tilapia, Tomato pomace

## Abstract

Several studies have predicted that developing countries will be unable to afford fish meal as a primary aquafeed protein source in the future. This experiment evaluated the effect of replacing fish meal (FM) with poultry by-product meal (PBM), tomato pomace, and azolla growth, physiological, and histological parameters, besides their economic impact on Nile Tilapia (*Oreochromis niloticus*). A total of 270 fish were randomly allocated to 6 groups (initial weight average was 12.08 g). The groups are as follows: a fish meal-based diet (T_c_), a poultry by-products meal-based diet (T_p_), a T. pomace-based diet (T_t_), T_t_ supplemented with Natuzyme (T_te_), an azolla-based diet (T_a_), and T_a_ supplemented with Natuzyme (T_ae_). Regarding the growth performance T_p_, T_a_, and T_ae_ were secondary to T_c_ and followed by T_t_ and T_te_. The hematological and biochemical indicators didn’t differ among groups. The Azolla-based groups had the lowest hepatosomatic index and the highest carcass fat and protein. Glutathione and interleukin-10 were higher in T.pomace and azolla enzyme-based diets. Malondialdehyde and tumor necrosis factor-alpha were lower in T_t_, T_te_ and T_ae_. Standard histological findings were observed in the spleen, kidney, and liver among groups. T_p_, T_a_, and T_ae_ significantly showed the highest absorption surface area (ASA) compared to T_t_ and T_te_. The photomicrographs demonstrated negative expression of nuclear factor kappa B (NF-κB) in all treatments. T_p_ outperformed T_c_ 45% economically, and azolla matched PBM. It could be concluded that Azolla serves as a plant-based alternative. PBM represents a sustainable, cost-effective replacement for FM, reducing reliance on conventional marine-alternative proteins.

## Introduction

Fish contribute to over 20% of global animal production and are a key protein source for around 3 billion people worldwide. Moreover, by 2030, fish produced worldwide through aquaculture is expected to account for nearly two-thirds of the world’s total fish consumption for food (FAO 2020). Nile tilapia (*Oreochromis niloticus*) is the second most farmed fish worldwide due to its ability to tolerate a wide variety of conditions compared to other species. Its popularity is attributed to its affordable cost and favorable nutritional benefits (Suárez-Puerto et al. 2023). Additionally, Nile tilapia is the most cultured and widely distributed fish in the Egyptian market, known for its pleasant taste and low price relative to other fish (Sherif 2021).

Fish meal (FM) is considered the chief protein source in tilapia feed as it contains a high content of highly digestible, balanced and essential amino acids in addition to fatty acids, minerals, and vitamins (Hardy 2010). Fish meal (FM) and soybean meal (SBM) are conventional protein sources in Tilapia diets and have significantly contributed to the advancement of aquaculture. Also, FM prices increased dramatically due to several climatic changes and geopolitical crises, resulting in severe price fluctuations. These fluctuations are attributed to the uncertainty of providing fishery supplies and affect supply chains, which makes FM substitution a crucial matter (FAO 2022). In the near past, the cost of SBM has increased because of the rising demand for soybeans due to competing uses for both human food and livestock feed markets (Glencross et al. 2023).

A viable substitute protein source for aquafeed should be nutrient-dense, easily accessible, and available in sufficient quantities to satisfy demand (a sustainable and nutritious source) (Salter and Lopez-Viso 2021).

Fish meal in the diets of Fingerling diets could be replaced with Poultry by-products meal (PBM) without compromising growth performance (Hernández et al. 2010). Up to 100% of FM can be replaced with PBM without negatively impacting the growth of tilapias (Yones and Metwalli 2015). PBM has a slightly lower crude protein level than FM. PBM is widely recognized to have a similar nutritional composition to FM (58–65% protein, 12–15% lipid). The source of PBM and manufacturing practices affect the final product quality (Hill et al. 2019). The amino acid profile of PBM is very similar to FM, but has a lower content of lysine and methionine (Gupta et al. 2020).

Egypt is considered the fifth-largest tomato-producing country in the world (Mahmoud and Osman 2023). Tomato pomace (T. pomace) is a wet waste product of industrial tomato processing. It comprises roughly 5–30% (w/w) of the original raw material and comprises peel, pulp, and seeds (Saini et al. 2018). Accordingly, food companies worldwide produce between 5.4 and 9.0 million tons of T. pomace annually (Lu et al. 2019). Crude protein makes up 15–25% of T. pomace on a dry matter basis, while NDF (Neutral detergent fibers) may reach up to 50% (Del Valle et al. 2006). T. pomace is considered as phenolic compounds, lycopene, and β-carotene, which are components of functional foods (Borycka 2017). Lycopene is a powerful antioxidant and anti-inflammatory natural compound that also exerts protective effects on cardiac tissues and helps in mitigating various metabolic syndromes (Ferreira-Santos et al. 2020). In the same context, the phenolic acids content positively affects the redox status (Fuentes et al. 2013). However, Nile tilapia could better utilize T. pomace up to 15% of the diet when combined with exogenous enzymes or amino acids (Hafez et al. 2024).

Aquatic plants are one of the most promising alternative feedstuffs besides terrestrial and plant by-products. Azolla is widely cultivated in Egyptian water systems due to its competitive advantages. Azolla replicates in 2–5 days, so it is one of the fastest-growing aquatic macrophytes (Taghi Ganji et al. 2005). The composition of Azolla (on dry matter basis) contains 3.0–4.4% crude fat, 13–35% crude protein, 9.7–23.8% ash, 5.6–15.2% cellulose, 9.8–17.9% hemicellulose, 9.3–34.8% lignin, (Yohana et al. 2023). Generally, many authors recommend that we can use Azolla as a source of protein to substitute FM at the rate of 20–30% without harmful effects on health, besides growth indices (Refaey et al. 2023).

However, several anti-nutritional elements in vegetable ingredients lessen nutrient digestion and absorption (Montoya-Camacho et al. 2019). Numerous studies indicate that exogenous enzymes can improve nutrient utilization by mitigating the adverse impacts of anti-nutritional components in plant feedstuffs, such as Natuzyme (Amer 2017).

A few studies discussed the effects of waste products from tomato-processing industries, specifically T. pomace (Amirkolaie et al. 2015; Hafez et al. 2024), and Azolla (Magouz et al. 2020; Refaey et al. 2023), as well as multienzyme supplementation (Zamini et al. 2014; Hlophe-Ginindza et al. 2016) on fish performance. Thus, this experiment aimed to explore the potential impacts of the PBM, T. pomace, and Azolla on Tilapia growth, feed utilization, hematological parameters, serum biochemical indices, antioxidant status, immunological parameters, organ histopathology, and nuclear factor kappa B (NF-κB) immunohistochemistry.

## Materials and methods

### Experimental conditions

The fish were obtained from the Fish Research Center SCU and were raised in concrete tanks. The experiment was conducted at the Aquarium Unit, Fish Farming and Technology Institute, SCU, Ismailia, Egypt. The experiment lasted for 12 weeks, preceded by two weeks of acclimatization period to the new environment (Glass aquaria). During the acclimatization period, all tilapias received the control diet at a rate of 3% of their body weight (Ali et al. 2025). The diet was divided into two meals daily at 10 a.m. and 4 p.m. (El-Naggar et al. 2021; Kirimi et al. 2023). Water temperature was measured daily using a standard thermometer and maintained at (27 ± 2℃), and water pH was measured weekly and maintained at (7.4–7.6) using a Waterproof pH-Temperature Pocket Tester with Electrode (ADWA AD11) (Ibrahim et al. 2024). These water parameters (temperature and pH) were maintained within the suitable range for tilapia habitats, as reported by Boyd and Tucker (2012). Dead fish were recorded and removed.

### Diets preparation

The fish meal was obtained from the “Aller Aqua” factory, 6th of October City, Egypt. Tomato pomace was provided by the " Shams Group Company”, Wadi Al Molak region, Ismailia, Egypt. It was air-dried and flipped 3 times a day to prevent spoilage (Abedalhammed et al. 2020). Azolla was acquired from a local farm in Fayoum governorate. It was dried away from direct sunlight for 3 weeks in a well-ventilated place, with continuous stirring (Naji and Alzurfi 2023). Natuzyme is a multienzyme that was produced by " Bioproton Europe Oy (Finland)”.Six isonitrogenous and isocaloric diets were formulated containing 33% CP, and a gross energy of 4588.91 Kcal/kg diet (Guo et al. 2011). The diets were processed into 2.5-mm-diameter pellets using a flat-die pelleting machine (Model KL-120, Laizhou Chengda Machinery Co., Ltd., Shandong, China).

### Experimental design

After acclimatization, 270 apparently healthy fish with an average body weight of 12.08 g were randomly allocated into 6 equal groups, each containing 3 replicates (15 fish/ replicate). Each replicate consisted of a glass aquarium (80 × 40 × 45 cm) stocked with 15 fish. The experimental groups were summarized as follows T_c_: received a control diet that included fish meal as the main source of animal protein. T_p_: received a diet based on poultry by-product meal (PBM) instead of fish meal. T_t_: received a PBM-based diet with TP to partially replace soybean meal (SBM), corn, and wheat flour. T_te_: the same as the T_t_ group, but with the addition of Natuzyme. T_a_: received a PBM-based diet with azolla to partially replace SBM, corn, and wheat flour. T_ae_: the same as the T_a_ group, but with the addition of Natuzyme.

### Chemical analysis of the ingredients and diets

Proximate chemical analysis for crude protein determination in the feed ingredients and basal diet for moisture, crude protein, ether extract, crude fiber, and ash contents, followed the official methods of AOAC (2005). Chemical analysis is provided in Table 1.

**Table 1:** The determined composition and calculated values of the different experimental diets (%)

Group Ingredients (%)		T_c_			T_*p*_	T_t_	T_te_	T_a_	T_ae_
Fish meal		21.05			-	-	-	-	-
PBM		-			23.22	26.03	26.03	28.52	28.52
Corn gluten		6			6	6	6	6	6
Wheat Flour		20.68			20.48	15	15	15	15
SBM		25			25	15	15	15	15
Yellow corn		16			16	10	10	10	10
Sunflower oil		8.28			6.30	7.68	7.68	9.39	9.39
TP		-			-	17.29	17.29	-	-
Azolla		-			-	-	-	13.10	13.10
Vitamin and mineral premix*		2.87			2.92	3	3	2.96	2.96
Limestone		0.13			-	-	-	-	-
Vitamin C (mg/kg)		50			50	50	50	50	50
L-Lysine (purity 99%)		-			0.02				
DL- Methionine (purity 99%)		-			0.06			0.04	0.04
Natuzyme g/ Kg diet		-			-	-	0.35		0.35
Calculated Values									
Crude protein %**		33			33	33	33	33	33
Gross energy (Kcal)**		4588.91			4588.9	4588.94	4588.94	4588.94	4588.94
Calcium %***		0.7			0.84	0.93	0.93	1.16	1.16
Phosphorus %***		0.61			0.6	0.64	0.64	0.80	0.80
Lysine % ***		2.33			1.6	1.88	1.88	1.61	1.61
Methionine% ***		1.00			0.7	0.71	0.71	0.7	0.7
Chemical composition of TP and azolla ****	**Moisture %**	**Gross energy** **(Kcal/Kg)**	**Crude protein %**	**Crude fiber %**	**Crude lipid%**	**Ash%**		**NFE%**	
TP	6.39	3227.70	20.06	30.68	9.46	5.31		28.1	
Azolla	8.75	1977.23	14.22	25.42	0.92	25.11		25.61	

T_c_ (Control, Fish meal based- diet), T_p_ (Poultry by-product meal based-diet), T_t_ (Tomato pomace based-diet ), T_te_ (Tomato pomace with enzymes based-diet), T_a_ (Azolla based-diet), T_ae_ (Azolla with enzymes based-diet)

*Each 500 g of vitamins premix contained 12.5 million IU of vitamin A, 50,000 mg of vitamin E, and 5 million IU of vitamin D_3_. 1000 mg folic acid, 2000 mg B_1_ and 5500 mg B_2_; 2500 mg B_6_ and 20 mg B_12_; 40,000 mg niacin; 100 mg biotin; 12,000 mg pantothenic acid, 3500 mg vitamin K_3_, 500 mg BHT, calcium carbonates up to 500 g. Each 500 g of minerals premix contained iron (45 g), zinc (75 g), copper (5 g), iodate (1.3 g), cobalt (0.1 g), manganese (80 g), selenium (0.3 g), calcium carbonates up to 500 g

**Requirement of energy and CP for Nile Tilapia from Guo et al. (2011).Gross Energy was calculated as 23.9, 39.8, and 17.6 kJ/g for protein, lipid, and NFE, respectively (Schulz et al. 2005), the conversion factors for joules and calories are: 1 kJ = 0.239 kcal; and 1 kcal = 4.184 kJ (Maclean et al. 2003)

NFE = 100- (% crude protein + % crude lipid + % ash+ % crude fiber+ moisture %)

***The amino acid, calcium, and phosphorus requirements were obtained from (NRC 2011)

**** The chemical composition of TP, and azolla were conducted at Nutri Lab, Tanta, Egypt according to the official standards methods for these analyses provided in (AOAC 2005)

### Growth performance measurements

To measure the body weight development in the different groups, fish from each aquarium were batch weighed using an electronic balance every 2 weeks. The following parameters were measured to assess the growth trial:

1. Mean fish weight = Total weight of tilapias in each replicate / Total number of fish in the replicate.
2. Weight gain (WG) = Mean body weight at the end (g) - Mean body weight at the beginning (g).
3. Average daily gain (g/day) = WG / Experimental periods (days).
4. Weigh gain percentage = 100 × (Mean body weight at the end (g) - Mean body weight at the beginning (g) / Initial body weight.
5. Specific growth rate (SGR, %/day) = 100 × [(ln final body weight − ln initial body weight) / time (days)] **(**Wu et al. 2023).
6. (Feed intake g/d/fish) = Daily feed intake/ Number of fish.
7. Feed conversion ratio = Feedstuff consumption (g) / WG (g).
8. Feed conversion efficiency = WG (g)/ Feedstuff consumption (g).
9. Protein efficiency ratio = WG (g) / Protein consumption (g).

### Sampling

At the end of the feeding trial, fish were fasted for 24 h to ensure gut evacuation. Two fish per replicate were randomly selected and anesthetized with clove oil solution (Healthy Zone^®^, 30 mg/L; clove oil: ethanol, 1:9). Blood samples (Approximately 0.5 ml) were collected from the caudal vein; one portion was treated with 10% EDTA for hematological analysis, while the other was centrifuged (3000 rpm, 15 min) to obtain serum for biochemical assays. Serum samples were stored at -20 °C until use. Additionally, one fish per aquarium was collected and stored at -20 °C for whole-body composition analysis.

Muscle and liver tissues were homogenized in phosphate-buffered saline (PBS, pH 7.4). Muscle supernatants were obtained by centrifugation (2,500 rpm, 10 min) for redox state analysis (Yang et al. 2023). Liver homogenates underwent two freeze-thaw cycles followed by centrifugation (5,000 × g, 5 min, 4 °C) to determine TNF-α and IL-10 levels. For histopathology and immunohistochemistry, intestine, liver, kidney, and spleen samples from O. niloticus were fixed in 10% buffered formalin for 24 h (Mohammady et al. 2021).

### Body composition

The stored fish was thawed to undergo proximate analysis according to **(**AOAC 2005**)** method.

### Carcass traits (Body condition indices)

1. Dressing percentage (Body without viscera but containing head) = 100× (Dressed carcass weight / Live weight) (Dias Trombeta et al. 2023).
2. Hepatosomatic index% = 100 × (Total liver weight / Body weight).
3. Viscerosomatic index% = 100 × (Internal organs weight / Body weight).

### Survival rate (SR%)

Survival rate = 100 × (Final number of tilapias at the end of the trial/ Initial number).

### Hematological parameters

The total RBCs and WBCs were detected manually with a hemocytometer chamber using Natt and Herrick’s solution as a diluent stain (Stoskopf 1993).

The hematocrit level was determined using the microhematocrit method, as described by Dawood et al. (2023) and Phinyo et al. (2024). Hemoglobin concentration was determined by the formation of cyanmethemoglobin using Drabkin’s reagent kits as a colorimetric method (Diamond Diagnostic, Egypt. The mean corpuscular volume (MCV), the mean corpuscular hemoglobin (MCH), and the mean corpuscular hemoglobin concentration (MCHC) were calculated as mentioned by Ali et al. (2025). DLC was measured according to Sattanathan et al. (2024) using Giemsa stain.

### Serum metabolites

Serum biochemical parameters were determined using a semi-automatic chemistry analyzer (Mindray, India). Liver enzyme activities, including alanine aminotransferase (ALT), aspartate aminotransferase (AST), and alkaline phosphatase (ALP), were measured according to Tietz (1987). Kidney function markers (urea and creatinine) and lipid profile components, including total cholesterol, triglycerides, and high-density lipoprotein (HDL), were determined using commercial kits (BioMed Diagnostic and Spectrum-Diagnostics, Egypt) following standard protocols (Gottfried and Rosenberg 1973; Allain et al. 1974; Henry et al. 1974; Lopes-Virella et al. 1977). Total protein and albumin were detected colorimetrically (Doumas and Biggs 1953). Globulin was calculated as the difference between total protein and albumin, while low-density lipoprotein (LDL) and very low-density lipoprotein (VLDL) were determined according to (Assmann et al. 1984); Friedewald et al. (1972), respectively.

### The redox status of Nile tilapia muscle

The Muscle Glutathione (GSH) assay was performed using ELISA kits as mentioned in (Baker et al. 1990; Pastore et al. 2001). The Malondialdehyde (MDA) ELISA test was done as mentioned by Hogberg et al. (1974) and Botsoglou et al. (1994). We used “Sun Long Biotech Co., LTD (GSH) ELISA Kits” and “Afg Bioscience Fish MDA ELISA Kits”.

### Liver cytokines

All procedures followed the instructions of Sun Long Biotech Co., Ltd., China. The catalogue numbers were Fish TNF-α: Cat. No., SL0055FI and Fish IL-10: Cat No., QS0059FI, SL0043Ch.This assay was performed as mentioned by Carswell et al. (1975), and Saraiva and O’Garra (2010).

### Histopathology

Tissue specimens were cleared in xylene and embedded in paraffin, and stained according to Suvarna et al. (2018). For Intestinal histomorphometry, Intestinal villi length, intestinal villi width, and absorption surface area (ASA) were inspected. ASA (mm^2^) = villus height x villus width (Mohammady et al. 2021).

### Immunohistochemical study

Liver sections underwent microwave-based antigen retrieval to unmask epitopes. Immunohistochemical staining was performed using a primary antibody against NF-κB p65 (Santa Cruz Biotechnology, Germany, sc-8008; 1:100 dilution) and a goat anti-mouse IgG secondary antibody (Invitrogen, USA, 318000; 1:600 dilution). Following hematoxylin counterstaining, slides were digitized using an Olympus digital camera (LC20) mounted on an Olympus BX-50 microscope (Japan) with a 40X objective (Cattoretti et al. 1993).

### Economic evaluation

According to Ibrahim et al. (2022), economic evaluation criteria were estimated. The total cost included costs such as feed, fingerlings, and additives.

Net profit (L.E/fish) = selling costs − total costs.

Economic efficiency% = Net profit / Total feed cost ×100.

### Statistical analysis

The analysis was done using a one-way analysis of variance (ANOVA). In this trial, Duncan’s multiple range test was employed as a post hoc test to detect significant differences among the groups (Duncan 1955). All procedures and statistical tests were conducted with the assistance of SPSS software, version 25.

## Results

### Growth performance, feed utilization, and survival rate

It was revealed that the fish fed on a fish meal-based diet showed significantly superior performance in final body weight, weight gain, and specific growth rate compared to all other treatments (*P* < 0.05), followed by the group fed on poultry by-product meal (T_p_ ) and azolla-based-diet groups (T_a_ and T_ae_). They were significantly improved over tomato pomace-based diet groups (T_t_ and T_te_). T_c_ was statistically higher than other groups (*P* < 0.05), in terms of feed efficiency, including feed conversion ratio, feed conversion efficiency (FCR), and protein efficiency ratio. Groups T_p_, T_a_ and T_ae_ were significantly greater than T_t_ and T_te_ in terms of feed efficiency (*P* < 0.05). Feed intake was higher in the control group compared to all groups (*P < 0.05*), followed by T_p_. All groups maintained high survival rates (> 84%), with no statistically significant differences among groups, indicating that none of the diets negatively affected fish health or viability (Table 2).

**Table 2:** Overall growth performance, feed utilization, and survival rate of Nile tilapia in different groups at the end of the experiment

Group	T_c_	T_*p*_	T_t_	T_te_	T_a_	T_ae_
Initial Weight (g/ fish)	12.04 ± 0.06 ^a^	12.09 ± 0.06 ^a^	12.04 ± 0.08 ^a^	12.13 ± 0.10 ^a^	12.11 ± 0.13 ^a^	12.04 ± 0.08 ^a^
Final Weight (g/ fish)	45.31 ± 0.97 ^a^	35.83 ± 2.47 ^b^	26.22 ± 1.20^cd^	25.73 ± 0.45^d^	31.30 ± 1.87 ^b^	30.85 ± 1.58^bc^
Weight gain (g/ fish)	33.27 ± 0.95 ^a^	23.75 ± 2.51^b^	14.18 ± 1.12^cd^	13.60 ± 0.47^d^	19.19 ± 2.00^bc^	18.81 ± 1.62^bc^
ADG (g/ fish)	0.40 ± 0.01^a^	0.28 ± 0.03 ^b^	0.17 ± 0.01 ^cd^	0.16 ± 0.01 ^d^	0.23 ± 0.02 ^bc^	0.22 ± 0.02 ^c^
Weight gain%	276.21 ± 7.50 ^a^	196.64 ± 21.59^b^	117.60 ± 8.46^cd^	112.10 ± 4.28 ^d^	158.83 ± 18.07 ^bc^	156.30 ± 14.17 ^bc^
DFI (Per Fish)	0.68 ± 0.02 ^a^	0.62 ± 0.02 ^b^	0.51 ± 0.02 ^c^	0.50 ± 0.004 ^c^	0.54 ± 0.01 ^c^	0.55 ± 0.02 ^c^
FI (g/fish/day)	57.09 ± 1.33 ^a^	52.37 ± 1.75^b^	42.71 ± 1.66^c^	41.67 ± 0.43 ^c^	45.27 ± 1.03 ^c^	45.83 ± 1.53 ^c^
SGR (%/ day)	1.58 ± 0.02 ^a^	1.29 ± 0.08 ^b^	0.92 ± 0.05 ^c^	0.89 ± 0.02 ^c^	1.13 ± 0.09 ^b^	1.12 ± 0.06 ^b^
FCR	1.72 ± 0.05 ^c^	2.24 ± 0.15 ^b^	3.03 ± 0.12 ^a^	3.07 ± 0.14 ^a^	2.41 ± 0.26 ^b^	2.46 ± 0.14 ^b^
FCE	0.58 ± 0.02^a^	0.45 ± 0.03^b^	0.33 ± 0.01 ^c^	0.33 ± 0.01 ^c^	0.42 ± 0.05^b^	0.41 ± 0.02^bc^
PER	1.77 ± 0.05 ^a^	1.37 ± 0.10 ^b^	1.00 ± 0.04 ^c^	1.00 ± 0.04 ^c^	1.29 ± 0.14 ^b^	1.24 ± 0.07^bc^
Survival rate (%)	100.00 ± 0.00 ^a^	84.33 ± 5.93^a^	86.67 ± 3.76^a^	93.33 ± 3.76^a^	82.22 ± 8.89 ^a^	91.10 ± 5.88^a^

Data are expressed as mean ± SE, standard error from triplicate

Different superscripts (a-d) in the same row indicate a significant difference (*P* < 0.05)

(ADG= average daily gain, DFI= daily feed intake, FI= Feed intake, SGR= specific growth rate, FCR= feed conversion ratio, FCE= feed conversion efficiency, PER = protein efficiency ratio.)

### Body condition indices

Live body weight, dressing weight, liver weight, and visceral weight were significantly higher in the T_c_ group compared to the other groups (*P* < 0.05). There were no statistical differences among the groups in dressing percentage and visceral index (*P* ˃ 0.05). The T_c_ group showed higher HSI either significantly with T_t_, T_a_ and T_ae_ groups, or numerically to T_p_ and T_te_ (Table 3).

**Table 3:** Body condition indices of Nile tilapia in different groups at the end of the experiment

Group	T_c_	T_*p*_	T_t_	T_te_	T_a_	T_ae_
LBW	51.45 ± 3.67 ^a^	34.55 ± 3.46 ^b^	30.97 ± 2.16 ^b^	28.41 ± 1.36 ^b^	33.65 ± 2.72 ^b^	34.10 ± 2.40 ^b^
Dressing weight	44.40 ± 3.47 ^a^	29.72 ± 3.22 ^b^	26.32 ± 1.92 ^b^	24.80 ± 1.25 ^b^	29.17 ± 2.27 ^b^	29.36 ± 2.10 ^b^
Dressing (%)	86.05 ± 0.88 ^a^	85.70 ± 1.06 ^a^	84.95 ± 1.02 ^a^	87.21 ± 0.51 ^a^	86.78 ± 0.89 ^a^	86.05 ± 1.06 ^a^
Liver weight	2.01 ± 0.15 ^a^	1.04 ± 0.16 ^b^	0.85 ± 0.18 ^b^	0.81 ± 0.06 ^b^	0.95 ± 0.14 ^b^	0.89 ± 0.13 ^b^
HSI (%)	3.94 ± 0.27 ^a^	3.04 ± 0.50 ^ab^	2.64 ± 0.38 ^b^	2.89 ± 0.24 ^ab^	2.80 ± 0.34 ^b^	2.60 ± 0.33 ^b^
Visceral Weight	7.05 ± 0.42 ^a^	4.83 ± 0.36 ^b^	4.65 ± 0.42 ^b^	3.62 ± 0.17 ^b^	4.48 ± 0.55 ^b^	4.74 ± 0.51 ^b^
VI	13.94 ± 0.88 ^a^	14.30 ± 1.06 ^a^	15.05 ± 1.02 ^a^	12.79 ± 0.51 ^a^	13.22 ± 0.89 ^a^	13.95 ± 1.06 ^a^

Data are expressed as mean ± SE, standard error from triplicate

Different superscripts (a-b) in the same row indicate a significant difference (*P* < 0.05)

(LBW= Live body weight, HSI = hepatosomatic index, VI= visceral index)

### Chemical composition of the whole carcass

Moisture content was statistically higher in the T_c_ group than other experimental groups (*P* < 0.05). T_p_ and T_te_ significantly had higher moisture content than T_a_, T_ae,_ and T_te_ groups (*P* < 0.05). Azolla-based diet groups and T_te_ were superior to the T_c_ and T_p_ groups in crude protein percentage (*P* < 0.05). The Azolla group statistically achieved the highest crude fat content compared to the other groups (*P* < 0.05). Crude fat of T_te_ was significantly superior to the T_t_ and T_p,_ and the T_c_ group was the least. T_p_ had the lowest ash percentage compared to other groups, followed by T_t,_ then T_a_, T_c_ and T_te_ (Table 4).

**Table 4:** Proximate Chemical composition of whole carcass of Nile tilapia in different groups at the end of the experiment (on wet basis %)

Group	T_c_	T_*p*_	T_t_	T_te_	T_a_	T_ae_
Moisture	72.21 ± 0.06 ^a^	71.21 ± 0.06 ^b^	70.83 ± 0.40 ^c^	71.21 ± 0.01^b^	70.93 ± 0.03 ^c^	70.86 ± 0.04 ^c^
Crude Protein	21.00 ± 0.13 ^c^	21.05 ± 0.05 ^c^	21.38 ± 0.13^b^	21.65 ± 0.11^ab^	21.90 ± 0.06 ^a^	21.88 ± 0.07 ^a^
Crude fat	2.33 ± 0.06 ^d^	2.95 ± 0.04^c^	2.97 ± 0.05^c^	3.43 ± 0.04^b^	3.86 ± 0.06 ^a^	3.93 ± 0.04 ^a^
Ash	2.55 ± 0.06 ^ab^	1.70 ± 0.02 ^e^	1.90 ± 0.05^d^	2.44 ± 0.03 ^b^	2.13 ± 0.10 ^c^	2.66 ± 0.10 ^a^

Data are expressed as mean ± SE, standard error from triplicates

Different superscripts (^a-e^) in the same row indicate a significant difference *(P < 0.05)*

### Hematological parameters

Generally, our results revealed that there were no significant differences among groups in hematological indices (*P* ˃ 0.05). Except for the RBC count, T_te_ had a significantly lower value than the other groups (*P < 0.05)*. Also, T_te_ and T_ae_ achieved the lowest level of hemoglobin concentration (Table 5).

**Table 5:** Hematological parameters of Nile tilapia in different groups at the end of the experiment

Group	T_c_	T_*p*_	T_t_	T_te_	T_a_	T_ae_
RBCs x10^6^ (cells /µL)	2.83 ± 0.02 ^a^	2.7 ± 0.52 ^a^	2.19 ± 0.25^ab^	1.53 ± 0.28^b^	2.74 ± 0.28 ^a^	1.80 ± 0.38^ab^
PCV (%)	27.0 ± 1.53 ^a^	25.33 ± 2.91 ^a^	24.66 ± 1.20 ^a^	19.67 ± 2.84 ^a^	27.00 ± 2.08 ^a^	22.66 ± 2.4 ^a^
Hemoglobin (g/dL)	9.06 ± 0.73 ^a^	8.26 ± 1.34^ab^	7.93 ± 0.64^ab^	6.13 ± 0.59^b^	8.90 ± 0.24^ab^	6.20 ± 1.04^b^
MCV (µ^3^)	95.68 ± 6.15 ^a^	96.84 ± 7.66 ^a^	115.02 ± 11.46 ^a^	130.91 ± 15.38 ^a^	99.31 ± 6.18 ^a^	133.03 ± 18.04 ^a^
MCH (µ^3^)	32.10 ± 2.88 ^a^	31.37 ± 4.14 ^a^	36.56 ± 1.87 ^a^	41.42 ± 4.98 ^a^	33.11 ± 3.08 ^a^	36.12 ± 5.88 ^a^
MCHC (%)	33.60 ± 2.23 ^a^	32.32 ± 2.89 ^a^	32.07 ± 1.46 ^a^	32.39 ± 4.81 ^a^	33.28 ± 2.05 ^a^	27.83 ± 4.56 ^a^
WBCs x10^3^ cells /µL	81.33 ± 11.72 ^a^	86.67 ± 27.03 ^a^	71.33 ± 9.40 ^a^	75.33 ± 17.68 ^a^	79.66 ± 23.68 ^a^	65.33 ± 16.33 ^a^
Lymphocytes %	94.67 ± 2.33 ^a^	96.33 ± 0.67 ^a^	94.00 ± 1.15 ^a^	96.33 ± 0.33 ^a^	94.33 ± 0.88 ^a^	96.00 ± 1.15 ^a^
Neutrophils %	1.67 ± 0.67 ^a^	1.67 ± 0.33 ^a^	2.33 ± 0.88 ^a^	1.67 ± 0.67 ^a^	3.00 ± 0.58 ^a^	1.67 ± 0.67 ^a^
Monocytes %	3.33 ± 1.33 ^a^	2.00 ± 1.00 ^a^	3.67 ± 1.20 ^a^	2.00 ± 0.57 ^a^	2.67 ± 0.67 ^a^	2.33 ± 0.4 ^a^
Eosinophils %	0.33 ± 0.33 ^a^	0.00 ± 0.00 ^a^	0.00 ^a^	0.00 ^a^	0.00 ^a^	0.00 ^a^
Basophils %	0.33 ± 0.33 ^a^	0.00 ± 0.00 ^a^	0.00 ^a^	0.00 ^a^	0.00 ^a^	0.00 ^a^

Data are expressed as mean ± SE, standard error from triplicates

Different superscripts (^a-b^) in the same row indicate a significant difference *(P < 0.05)*

### Serum Metabolites

The data shown in Table 6. For the ALT test, T_ae_ was statistically higher than T_p_ (*P < 0.05*), with no significant differences within other groups. Besides, the T_te_ group showed high AST levels compared to T_c_. T_a_ had a high level of ALP either numerically with T_te_ or significantly with other groups. Though all detected values were considered within the normal range of serum biochemical parameters. There was no statistical difference among groups in the serum protein levels (*P* ˃ 0.05).

**Table 6:** Serum metabolites of Nile tilapia in different groups at the end of the experiment

Group	T_c_	T_*p*_	T_t_	T_te_	T_a_	T_ae_
ALT (U/L)	23.55 ± 0.90^ab^	21.68 ± 1.09^b^	23.15 ± 1.11^ab^	22.95 ± 1.19^ab^	23.68 ± 1.16^ab^	25.27 ± 0.87 ^a^
AST (U/L)	21.87 ± 1.07^b^	22.88 ± 1.02^ab^	24.17 ± 0.93^ab^	24.92 ± 1.15 ^a^	23.18 ± 0.73^ab^	22.8 ± 0.46^ab^
ALP (U/L)	10.37 ± 0.33^b^	10.97 ± 0.42^b^	10.48 ± 0.81^b^	11.18 ± 0.26^ab^	12.75 ± 0.83 ^a^	10.46 ± 0.31^b^
Creatinine (g/dl)	0.38 ± 0.05 ^b^	0.6 ± 0.03 ^a^	0.52 ± 0.05 ^a^	0.33 ± 0.04 ^b^	0.34 ± 0.03 ^b^	0.32 ± 0.03 ^b^
Urea (g/dl)	6.62 ± 0.24 ^a^	6.22 ± 0.34 ^a^	6.21 ± 0.29 ^a^	6.48 ± 0.27 ^a^	6.51 ± 0.24 ^a^	6.25 ± 0.17 ^a^
Total protein (g/dL)	6.02 ± 0.38 ^a^	6.33 ± 0.33 ^a^	6.53 ± 0.33 ^a^	5.42 ± 0.44 ^a^	5.43 ± 0.20 ^a^	5.92 ± 0.40 ^a^
Albumin (g/dL)	2.84 ± 0.15 ^a^	2.81 ± 0.12 ^a^	2.89 ± 0.24 ^a^	2.94 ± 0.19 ^a^	2.89 ± 0.14 ^a^	2.74 ± 0.12 ^a^
Globulin (g/dL)	3.18 ± 0.29 ^a^	3.52 ± 0.39 ^a^	3.64 ± 0.42 ^a^	2.47 ± 0.55 ^a^	2.55 ± 0.3 ^a^	3.18 ± 0.39 ^a^
A/G ratio	0.92 ± 0.07 ^a^	0.87 ± 0.13 ^a^	0.90 ± 0.19 ^a^	2.22 ± 1.10^a^	1.24 ± 0.2 ^a^	0.97 ± 0.174 ^a^
Cholesterol (mg/dl)	152.18 ± 4.18^abc^	143.03 ± 6.55^c^	152.43 ± 6.76^abc^	158.66 ± 3.89^ab^	160.58 ± 3.62 ^a^	143.83 ± 2.78^bc^
Triglyceride(mg/dl)	259.67 ± 14.57^ab^	270.11 ± 18.46^ab^	227.16 ± 18.81^b^	273.95 ± 14.88^ab^	313.26 ± 8.09 ^a^	252.08 ± 16.97^b^
HDL (mg/dl)	31.08 ± 2.78 ^a^	30.28 ± 1.87 ^a^	32.48 ± 2.49 ^a^	30.25 ± 2.1 ^a^	33.61 ± 1.49 ^a^	27.18 ± 0.87^a^
LDL (mg/dl)	69.16 ± 6.22 ^a^	58.72 ± 9.34 ^a^	74.51 ± 5.26 ^a^	73.63 ± 2.75 ^a^	64.31 ± 3.23 ^a^	66.22 ± 6.08^a^
VLDL (mg/dl)	51.93 ± 2.91^b^	54.02 ± 3.69^ab^	45.43 ± 3.76^b^	54.79 ± 2.98^ab^	62.65 ± 1.62 ^a^	50.41 ± 3.39^b^

Data are expressed as mean ± SE, standard error from triplicates

Different superscripts (^a-c^) in the same row indicate a significant difference *(P < 0.05)*

Creatinine assay results indicated that T_p_ and T_t_ were statistically greater than those of the other groups (*P < 0.05*). The Urea test did not significantly differ among groups (*P* ˃ 0.05).

T_c_, T_t_, T_ae_ and T_p_ showed a significantly decreasing level of total cholesterol compared to T_te_ and T_a_ groups (*P < 0.05*). T_c_, T_p,_ T_t,_ T_te_ and T_ae_ did not have significant changes in triglyceride and VLDL levels. The HDL and LDL results exhibited no significant difference among the experimental groups (*P* ˃ 0.05).

### The redox status of Nile tilapia muscle

**Fig. 1:**
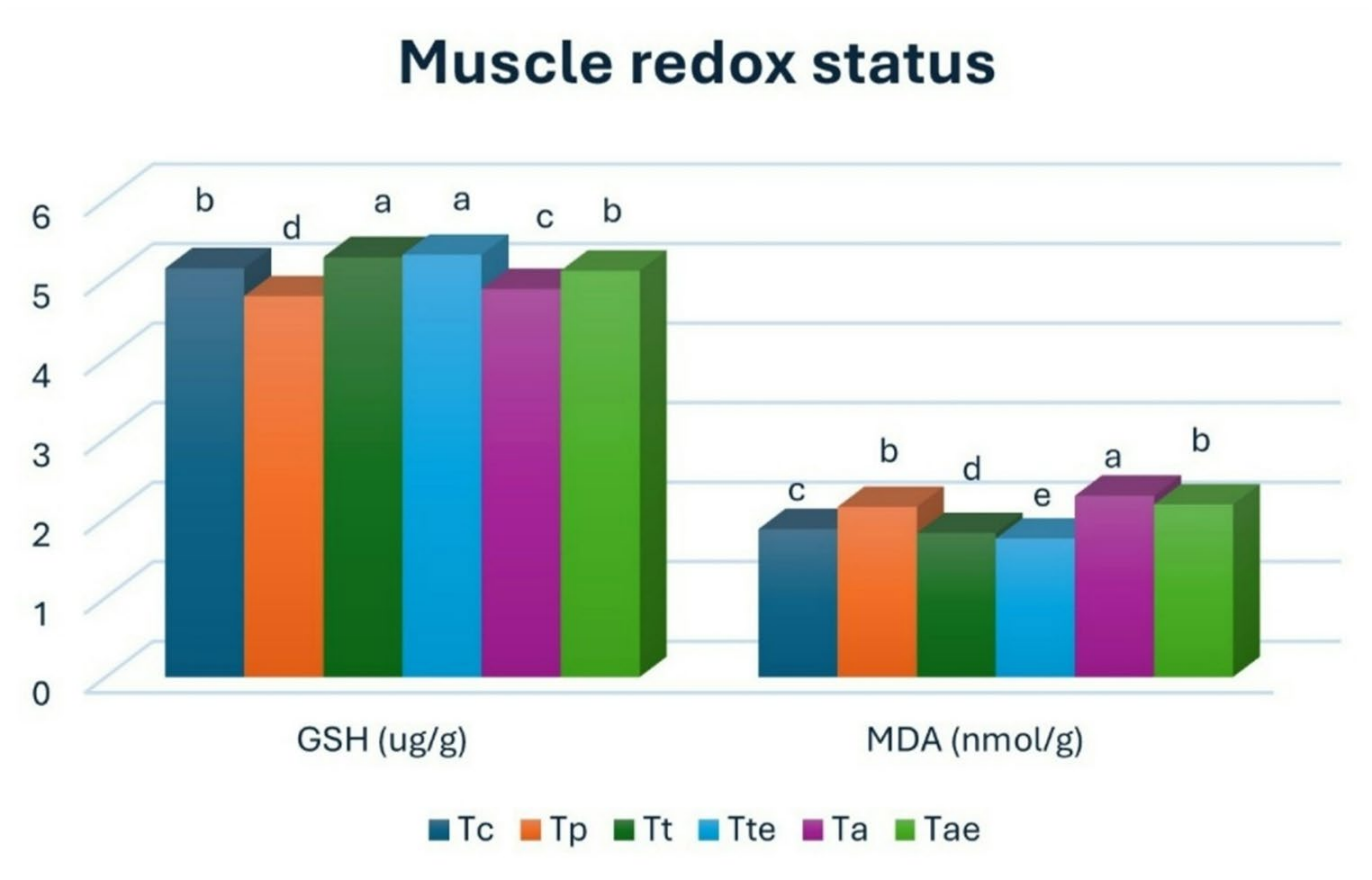
Muscle MDA (ug/g) and GSH (nmol/g) values of Nile tilapia in the different groups at the end of the experiment. Bars with different letters (a-e) indicate a significant difference *(P < 0.05)*

T_t_ and T_te_ revealed a significantly higher level of GSH, followed by T_c_ and T_ae_. T_p_ had the lowest level, then T_a_*(P < 0.05*). T_t_ and T_te_ had significant differences from the other groups; they had the lowest MDA level. The T_a_ group had the highest MDA level, followed by T_P_ and T_ae,_ then T_c_ (Fig. 1).

### Liver cytokines

**Fig. 2:**
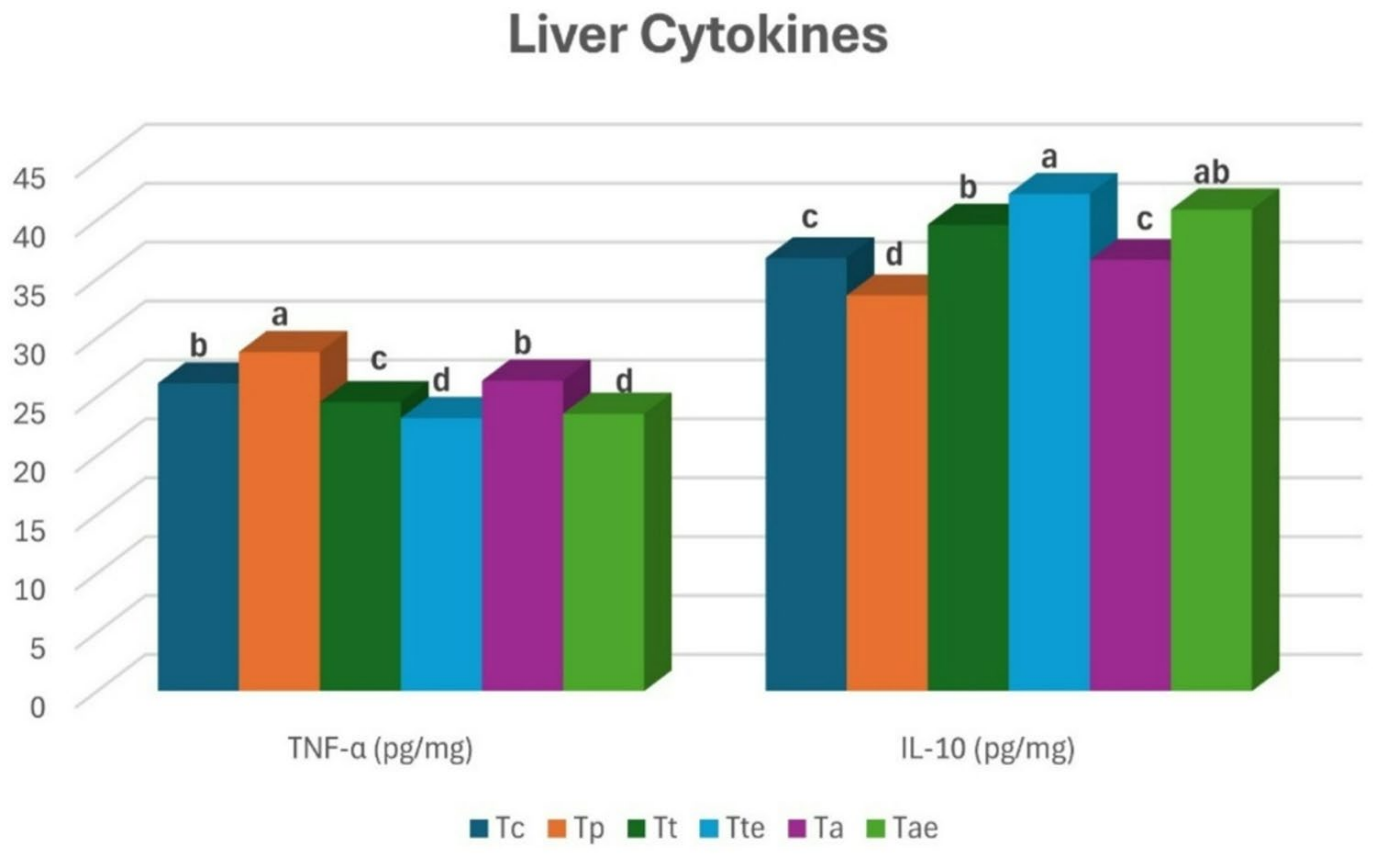
Liver TNF-α (a) and IL-10 levels of Nile tilapia in the different groups at the end of the experiment. Bars with different letters (a-d) indicate a significant difference *(P < 0.05)*

Liver TNF-α level was significantly decreased in T_te_ and T_ae,_ followed by T_t_, in comparison to other groups (T_c_, T_p_ and T_a_). Besides, T_te_ and T_ae_, followed by T_t_, significantly showed higher IL-10 levels compared to other groups (*P < 0.05*) (Fig. 2).

### Histopathology and intestinal histomorphometry

**Fig. 3:**
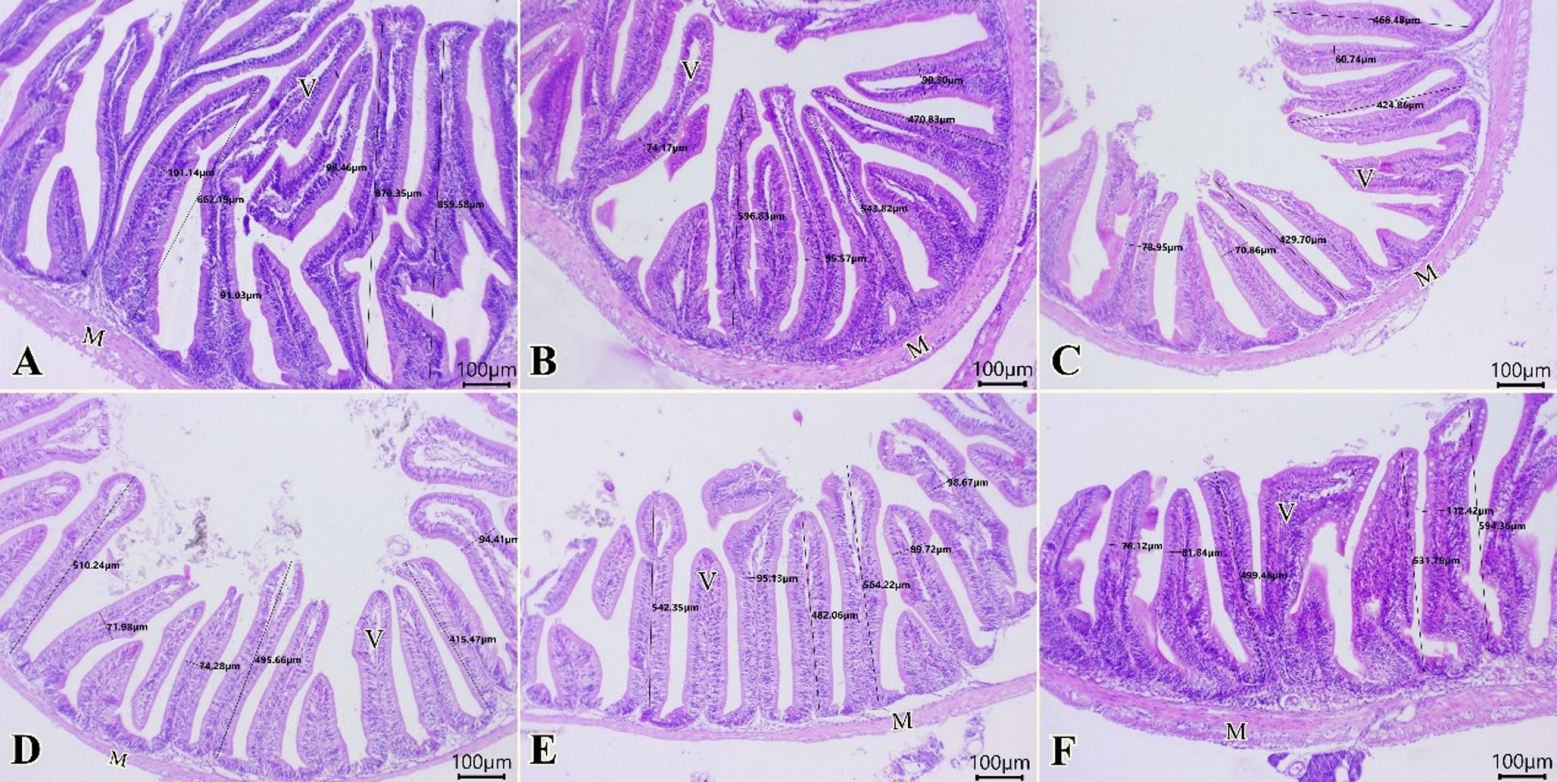
Photomicrograph of H&E-stained sections from the intestine of tilapia fish (Scale bar 100 μm) showing: **A**-**F**: Normal configurations of epithelial lining mucosal villi (V), submucosal layer, and muscular layer (M). The highest levels of intestinal parameters are at T_c_(A), then T_c_ (B), followed by T_ae_ (F)

The intestines from all examined groups (Fig. 3A-F) demonstrated normal configurations of epithelial lining, mucosal villi, submucosal layer, and muscular layer. The highest levels of intestinal parameters were seen in T_c_ (Fig. 3A), then Tp (Fig. 3B), followed by Tae (Fig. 3F). Table 7 illustrates the histo-morphometric features of the intestine. It was revealed that the Tc group showed the highest absorption surface area (ASA). Also, T_p_, T_a_, and T_ae_ significantly showed greater ASA compared to T_t_ and T_te_.

**Table 7:** Intestinal morphometry of Nile tilapia in different groups at the end of experiment

Group	T_c_	T_*p*_	T_t_	T_te_	T_a_	T_ae_
Villous length (µm)	797.37 ± 67.67 ^a^	541.83 ± 37.24 ^b^	441.01 ± 13.80 ^b^	473.79 ± 29.46^b^	529.54 ± 24.57 ^b^	541.87 ± 27.85 ^b^
Villous width (µm)	96.88 ± 3.02 ^a^	89.01 ± 7.44 ^ab^	70.18 ± 5.27^b^	80.22 ± 7.12 ^ab^	84.51 ± 8.07 ^ab^	85.46 ± 4.13 ^ab^
ASA * (mm	0.08 ± 0.003 ^a^	0.05 ± 0.003 ^b^	0.03 ± 0.001 ^d^	0.04 ± 0.000^cd^	0.04 ± 0.004 ^bcd^	0.05 ± 0.004 ^bc^

Data are expressed as mean ± SE, standard error from triplicates

Different superscripts (a-d) in the same row indicate a significant difference (*P* < 0.05)

* ASA = Absorption surface area

**Fig. 4:**
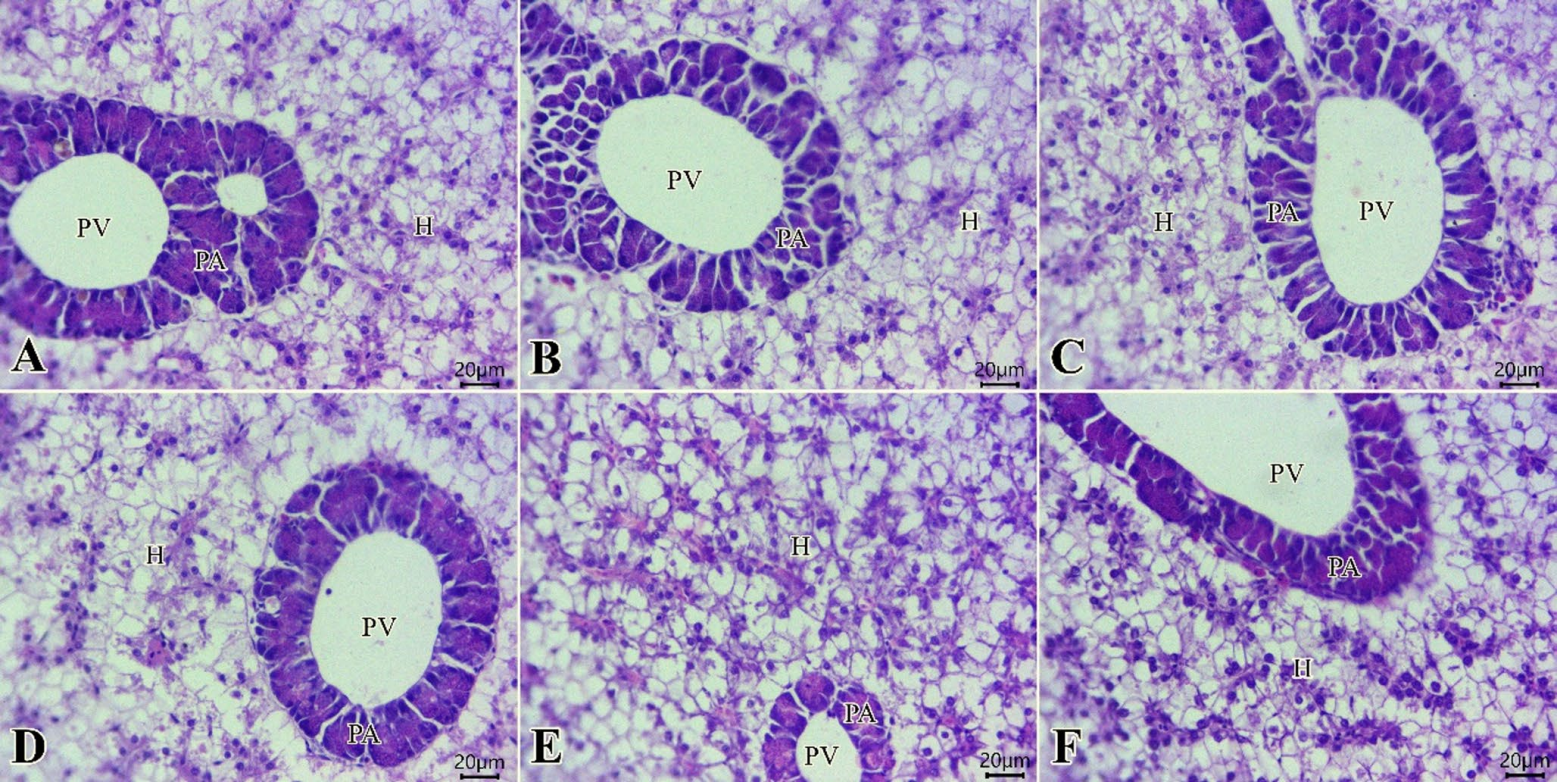
Photomicrograph of H&E-stained sections from the liver of tilapia fish (Scale bar 20 μm) showing: **A**-**F**: normal radially arranged hepatocytes (H), exocrine pancreatic acini (PA), and portal vein (PV) in all examined groups

Sections from the liver of all groups (Fig. 4A- F) exhibited normal radially arranged hepatocytes with narrow hepatic sinusoids. Further, groups of pancreatic cells were arranged in acinar forms around branches of the portal vein. Active apical granules within pancreatic acinar epithelium were commonly seen at T_c_ and T_p_.

**Fig. 5:**
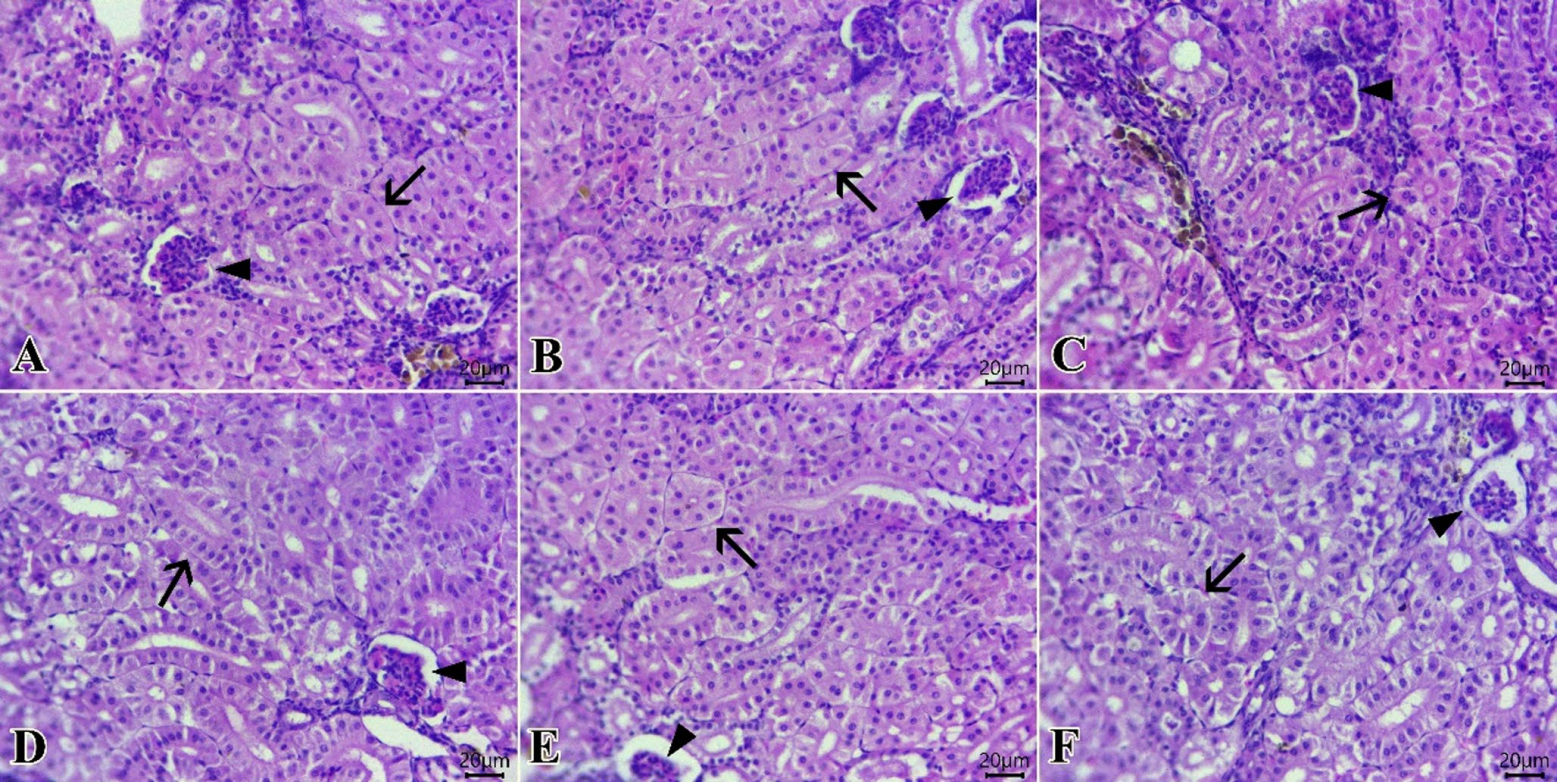
Photomicrograph of H&E-stained sections from the kidney of tilapia fish (Scale bar 20 μm) showing: **A**-**F**: normal morphology of renal tubules (arrows), glomeruli (arrowheads), and interstitial tissues in all examined groups

The kidneys of all groups (Fig. 5A-F) exhibited normal morphology of renal tubules, glomeruli, and interstitial tissues. Melano-macrophage centers were seen at some examined sections, primarily at T_t_ (Fig. 5C).

**Fig. 6:**
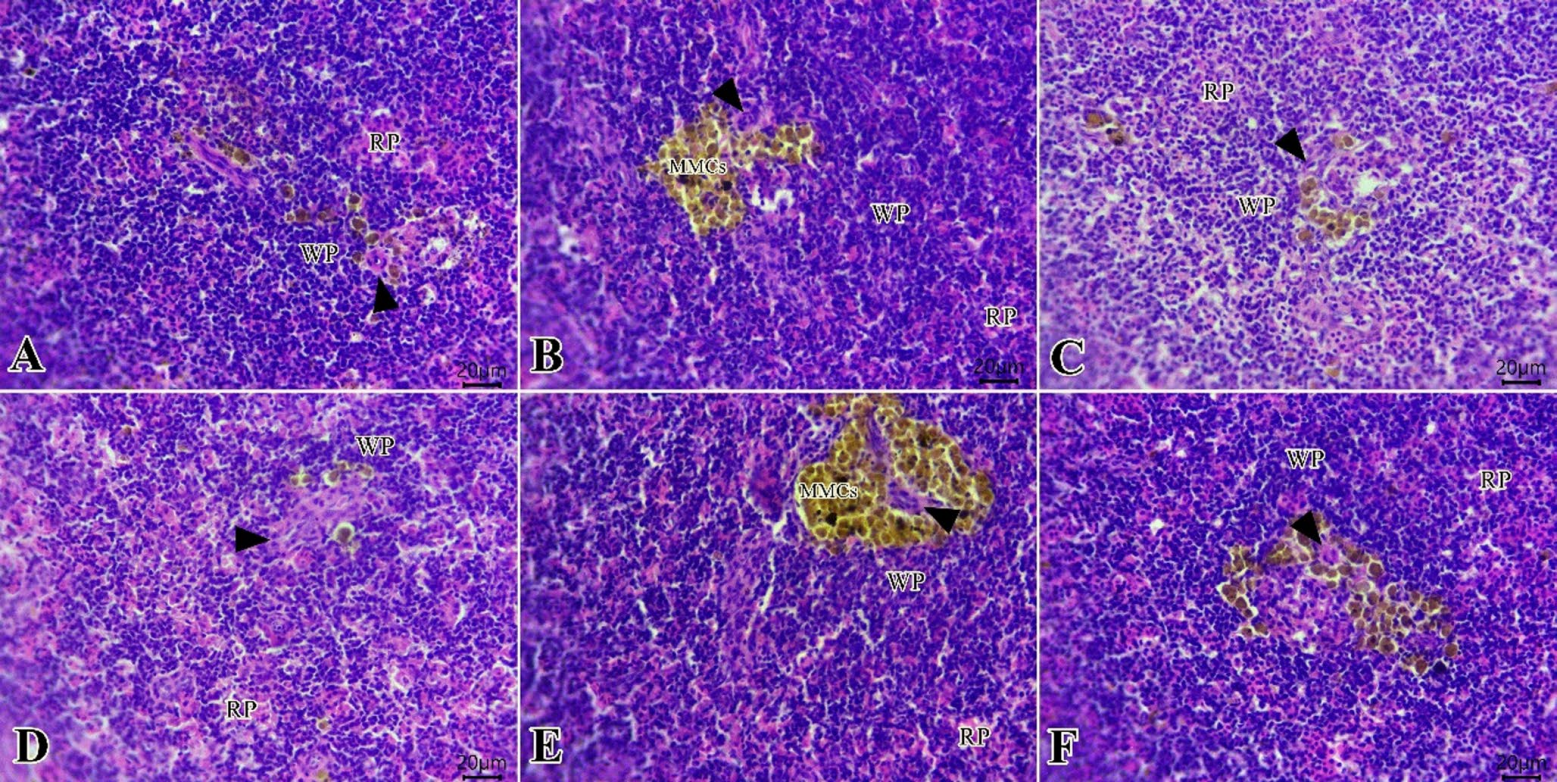
Photomicrograph of H&E stained sections from spleen of tilapia fish (Scale bar 20 μm) showing: **A**-**F**: normal cytoarchitectures of white pulp (WP) around the ellipsoid’s arterioles (arrowheads) with melanomacrophage deposits (MMCs) beside normal red pulp (RP) in all examined groups (G.1-G.6). High density of lymphoid elements at white pulp at T_c_ (A) and T_p_ (B) when compared with other groups

The spleen of all groups (Fig. 6A-F) revealed normal cytoarchitectures of white pulp around the ellipsoid’s arterioles with melanomacrophage deposits beside normal red pulp with reticular network, aggregations of lymphocytes, monocytes, and red blood cells. A high density of lymphoid elements at the white pulp was seen primarily at T_c_ and T_p_ when compared with other groups.

### Immunohistochemical findings

**Fig. 7:**
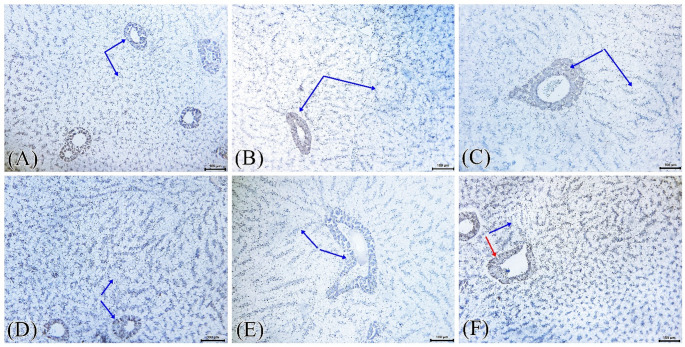
Immunohistochemical detection of NF-κB expression in hepatic tissue of Oreochromis niloticus across different dietary groups. photomicrographs showing predominantly negative NF-κB immunoreactivity (blue arrow) in hepatocytes and hepatopancreatic cells across all experimental groups: **A**- T_c_, **B**-T_p_, **C**- T_t_, **D**-T_te_, **E**- T_a,_ and **F**- T_ae_ ). Rare, weakly positive cells showing non-specific staining (red arrows). scale bar=100 μm

**Fig. 8:**
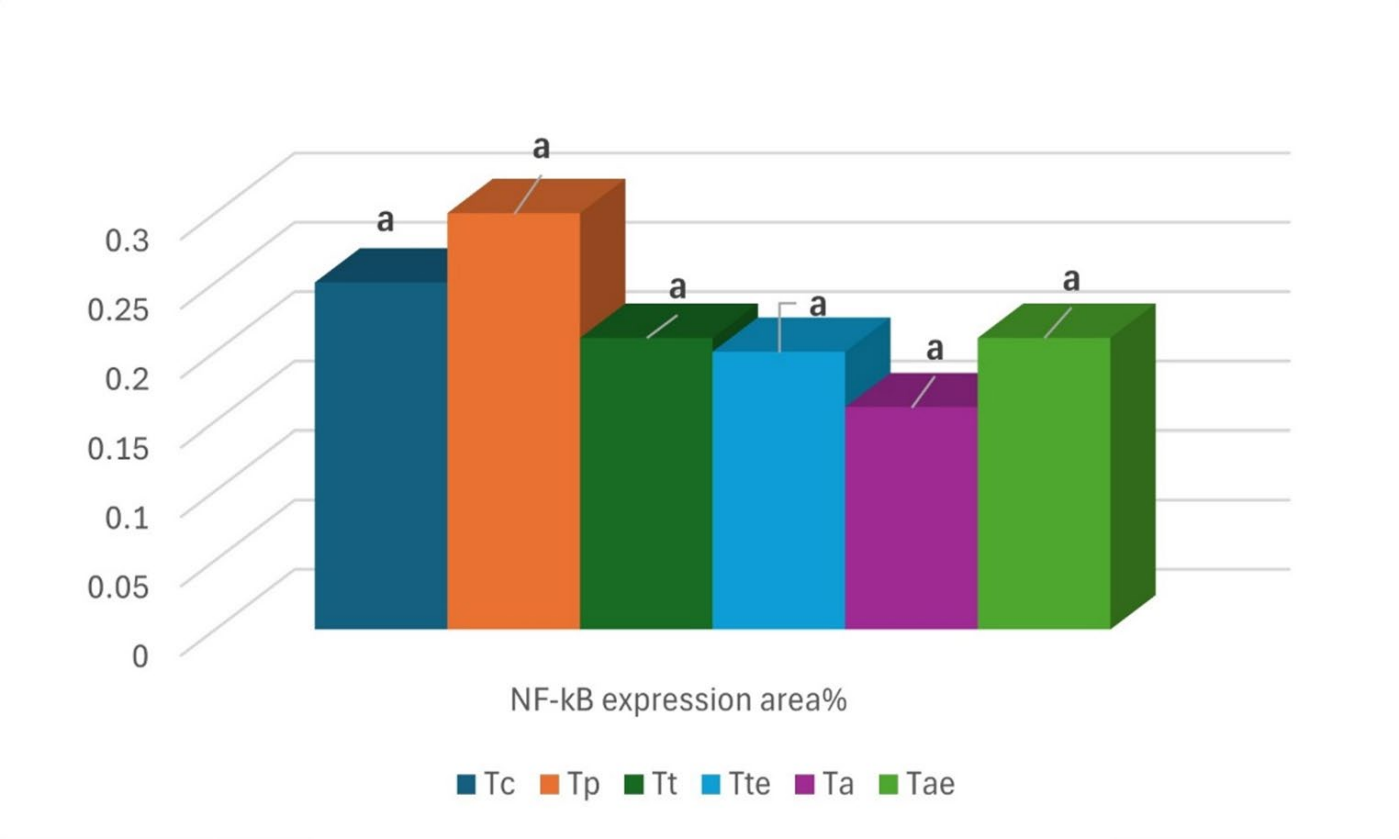
Demonstrates the percentages of expression of NK-_k_B in the hepatic tissue of all experimental groups

Examined immune-stained tissue sections from Nile tilapia revealed negative expression of the NF-_k_B marker in all the experimental groups, sparing very few unspecific weakly reactive cells that were seen in the hepatocytes and hepato-pancreatic cells of T_ae_. Negative cells were pointed out by dark blue arrows and positive cells by the red arrows (Fig. 7). NF-kB expression area% assay confirmed that there was no significant difference among the examined groups (P ˃ 0.05) (Fig. 8).

### Economic evaluation

T_c_, T_p_, T_a_ and T_ae_ achieved a significant maximum net profit compared to other groups *(P < 0.05*). T_p_ had the greatest economic efficiency, either significantly compared to T_t_ and or T_te_ or numerically to T_c_, T_a_ and T_ae_. Also, there were no significant differences detected in the economic efficiency percentage between the fish meal group and T_a_ and T_ae_ (Table 8).

**Table 8:** Economic efficiency in producing one kg of Nile tilapia fed different experimental diets

Group	T_c_	T_*p*_	T_t_	T_te_	T_a_	T_ae_
Cost of kg feed (LE) *	50.96	36.37	34.17	34.36	37.58	37.77
Purchase price (LE)	1.00	1.00	1.00	1.00	1.00	1.00
Final Body weight (Kg)	0.0453 ± 0.001 ^a^	0.0358 ± 0.002 ^b^	0.0262 ± 0.001^cd^	0.0257 ± 0.0004 ^d^	0.0313 ± 0.002^b^	0.0308 ± 0.002^bc^
Total Feed intake (kg/fish/	0.057 ± 0.001 ^a^	0.052 ± 0.003 ^b^	0.043 ± 0.002 ^c^	0.042 ± 0.001 ^c^	0.045 ± 0.001 ^c^	0.046 ± 0.001 ^c^
Feed cost/fish (L.E)	2.91 ± 0.07 ^a^	1.91 ± 0.06 ^b^	1.46 ± 0.06 ^d^	1.43 ± 0.01 ^d^	1.70 ± 0.04 ^c^	1.73 ± 0.06 ^c^
Total cost/fish (L.E) **	3.91 ± 0.07 ^a^	2.91 ± 0.06 ^b^	2.46 ± 0.06 ^d^	2.43 ± 0.01 ^d^	2.70 ± 0.04 ^c^	2.73 ± 0.06 ^c^
Selling price (L.E)	4.53 ± 0.1 ^a^	3.58 ± 0.25 ^b^	2.62 ± 0.12^cd^	2.58 ± 0.04 ^d^	3.13 ± 0.32^b^	3.09 ± 0.27 ^bc^
Net profit ***	0.62 ± 0.10 ^a^	0.68 ± 0.19 ^a^	0.16 ± 0.06 ^b^	0.14 ± 0.06 ^b^	0.43 ± 0.19 ^ab^	0.35 ± 0.11 ^ab^
Economic Efficiency ****	15.96 ± 2.79 ^ab^	23.14 ± 5.85 ^a^	6.51 ± 2.43 ^b^	5.84 ± 2.44 ^b^	15.88 ± 6.99 ^ab^	12.85 ± 3.76 ^ab^

Data are expressed as mean ± SE, standard error from triplicate

Different superscripts (a-d) in the same row indicate a significant difference (*P* < 0.05)

* = Based on local market price (LE/kg) of the following feed ingredients: (Fish meal 100, Soybean meal 26, Corn gluten 65, Yellow corn 12, Poultry meal 35, Azolla 20, Tomato pomace 2, Wheat flour 27, oil 89, Natuzyme 536, Premix 160, Lysine 60, Methionine 70, Vitamin C (100 gm) 35, Limestone 1 L.E ), selling price of 1 kg of tilapia = 100 L.E

** = Price / fish (L.E) + Feed cost / fish (L.E)

*** = Selling price (L.E.) – Total cost/ fish (L.E)

**** = Net profit/ Total cost/ fish×100

AOAC (2005) Official Methods of Analysis (15th Ed.). The Association of Official Analytical Chemist, Washington DC. 10.1002/0471740039.vec0284

Guo Y-X, Dong X-H, Tan B-P, Chi S-Y, Yang Q-H, Chen G, Zhang L (2011) Partial replacement of soybean meal by sesame meal in diets of juvenile Nile tilapia, Oreochromis niloticus L. Aquac Res 42 (9):1298–1307. 10.1111/j.1365-2109.2010.02718.x

Maclean W, Harnly J, Chen J, Chevassus-Agnes S, Gilani G, Livesey G, Warwick P Food energy–Methods of analysis and conversion factors. In: Food and agriculture organization of the united nations technical workshop report, 2003. vol 8. The Food and Agriculture Organization Rome, Italy,

NRC (2011) Nutrient Requirements of Fish and Shrimp. The National Academies Press, Washington, DC. 10.17226/13039

Schulz C, Knaus U, Wirth M, Rennert B (2005) Effects of varying dietary fatty acid profile on growth performance, fatty acid, body and tissue composition of juvenile pike perch (Sander lucioperca). Aquacult Nutr 11 (6):403–413. 10.1111/j.1365-2095.2005.00369.x

## Discussion

The growth indices, including WG and SGR, showed that T_p_, T_a_ and T_ae_ were secondary to T_c_ without a detrimental effect on these parameters. Feed utilization data, including FI, FCR, FCE, and PER, revealed similar findings, which assured these alternatives as promising feedstuffs. These observations agreed with further publications that verified PBM as the best, most economical, and commonly used animal protein source to substitute FM (Dawson et al. 2018). Azolla and T. pomace were used to replace other plant ingredients, like soybean meal and corn etc. (Ismail et al. 2022; Hafez et al. 2024; Aboseif et al. 2025). PBM had arisen as a viable substitute for FM in aquaculture production, due to high protein levels, lower ash content, strong acceptance by various species due to its palatability and attractiveness, being an excellent source of cholesterol and phospholipids, global availability, and lower cost in comparison to FM, as reported by Galkanda-Arachchige et al. (2020). Besides, it has a similar amino acid profile to FM (NRC 2011). Furthermore, Hernández et al. (2010), Yones and Metwalli (2015) and Mahmoud et al. (2023) confirmed the possibility of the total replacement of FM by PBM without harmful effects on fish growth in Nile tilapia. In our study, the azolla-based diets (13% of the total diet) had no adverse impact on growth data. This was in agreement with Magouz et al. (2020) and Refaey et al. (2023), who reported that azolla, up to 20% of the diet, had no adverse effect on growth. The growth rate was enhanced by good properties of Azolla, such as its acceptable crude protein content, EAA profile compared to other green fodder crops and most aquatic plants, besides having vitamins and carotenoid substances (Prasad et al. 2014). Mosha et al. (2020) and Lumsangkul et al. (2022) concluded that using more than 15% harmed growth parameters in Nile tilapia. Limitations of using azolla with high levels revolved around the high fiber content and ash, which hindered the digestibility of other nutrients (Refaey et al. 2023), besides having some antinutrients (Abd and Taha 2021).

T_t_ and T_te_ showed lesser growth performance due to their high fiber content. Tomato pomace, despite having a higher protein percentage than azolla, yielded lower results. It could be explained due to the poor digestibility of.

T. pomace protein in fish, which is highly affected by high fiber content, according to Amirkolaie et al. (2015) and Hafez et al. (2024) confirmed that the inclusion level of T. pomace should not exceed 15% of the total diet. On the other hand, Hussin et al. (2010) suggested that up to 26% of T. pomace could be included in the diet without adverse effects. Regarding enzymatic supplementation, the addition of 0.375 g/kg diet of Natuzyme did not significantly influence growth indices. This observation aligns with Amer (2017), who recommended an inclusion level of 1.5 g/kg diet for Nile tilapia. Similarly, Hlophe-Ginindza et al. (2016) advised a dosage of 0.5 g/kg diet for Mozambique tilapia (*Oreochromis mossambicus*).

The survival rate has proven that the local feed alternatives didn’t harm the health condition of Tilapia. All experimental groups didn’t differ significantly in dressing percentage (DP). These results reflected the yield of the edible part of the fish, which wasn’t affected by the alteration of traditional feedstuffs with local ones. These findings agreed with Emre et al. (2003) and Sevgİlİ and Ertürk (2004) about the effects of poultry meal on dressing percentage in mirror carp (*Cyprinus carpio*) and rainbow trout *(Oncorhynchus mykiss*), respectively. Soltan (2002) declared that using T. pomace at a 20% addition level or more didn’t affect the DP. However, Soltan et al. (2005) stated that using the same percentage of T. pomace in common carp significantly lowered the DP. The liver has a vital role in the metabolism of nutrients, compound detoxification, and protein synthesis, in addition to its immunological functions. Therefore, it is regarded as the key organ of fish that reflects their health and normal physiological functions. The hepatosomatic index (HSI), which assesses the liver’s relative size to body weight, and any change in this index is a sign of the liver’s response to various stressors, including dietary concerns (Matulic et al. 2020). T_p_ and T_te_ were at the same significance level as T_c_ in terms of HSI. The effect of PBM on HSI has been widely studied in Nile tilapia and other species (Fontinha et al. 2021; Mahmoud et al. 2023; Yu et al. 2023). Most authors agreed with our findings in the absence of a significant difference between the PBM-based diet and the control diet. However, Tang et al. (2025) revealed an elevation of HSI in mandarin fish (*Siniperca chuatsi*). Our investigations about T_a_ and T_ae_ agreed with Caruso et al. (2023), who referred to the decline of HSI after using azolla. On the contrary, Ismail et al. (2022) and Refaey et al. (2023) concluded that even the usage of azolla up to 30% of the total diet didn’t vary the HSI. The minimal effect of using azolla and T. pomace may be attributed to a change in hepatic metabolic activity or energy distribution, especially in high fiber diets (Villasante et al. 2025). The results ensured that there was no significant difference between groups in VSI. This was in line with the results of Aydin and Gümüş (2013), Fontinha et al. (2021) and Rawles et al. (2022) in terms of PBM inclusion. The effect of azolla on VSI was similar to (Ismail et al. 2022; Rahmah et al. 2022). Soltan (2002) who reported that using 28% of T. pomace didn’t alter the visceral weight.

In terms of carcass crude protein and fat percentage, T_a_ and T_ae_ were superior to the other groups. Also, Bharti et al. (2024) confirmed a direct relationship between increasing crude protein, crude fat, and the inclusion level of azolla in common carp (*Cyprinus carpio*). Refaey et al. (2023) confirmed that using 20% of azolla in the diet positively influenced the CP percent of the carcass. On the contrary, Magouz et al. (2020) reported that elevated levels of azolla didn’t affect the CP and crude fat in GIFT tilapia. Abou et al. (2011) and Ahmed et al. (2023) showed that the elevated levels of azolla inversely affected the CP and crude fat levels in Nile tilapia and common carp, respectively. In the same context, T_t_ and T_te_ were superior to T_c_. On the other hand, Hussin et al. (2010) ensured that T. pomace didn’t influence the CP and crude fat levels in tilapia. Soltan (2002) showed that T. pomace had decreased CP, crude fat in Nile tilapia. Salama et al. (2015) revealed that the addition of T. pomace influenced crude fat but didn’t affect CP in grass carp (*Ctenopharyngodon idella*). Also, crude fat of the whole fish carcass in T_p_ was superior to T_c_, without a difference between the two groups in CP content. Mahmoud et al. (2023) and Hernández et al. (2010) confirmed our findings about PBM. On the contrary, Yones and Metwalli (2015) reported that PBM didn’t affect either CP or crude fat. Natuzyme-supplemented groups had influenced the ash content in T_te_ and T_ae_. On the other hand, most studies revealed that there was no effect of using exogenous enzymes on the ash content of fish carcasses (Adeoye et al. 2016; Magalhães et al. 2016). This can be attributed to the different environmental conditions of the experiments, species differences, changes in the feed ingredients used, and even the type of exogenous enzymes.

In the same context, Bharti et al. (2024) revealed that the ash content had declined in the azolla groups compared to the control one. Nevertheless, Ahmed et al. (2023) declared that azolla didn’t alter the ash content of common carp. Sallam et al. (2024) reported that azolla had increased the ash content compared to the control diet in red tilapia (*Oreochromis spp.*). Inclusion of T. pomace in the diet decreased the ash content compared to the control diet, which was similar to the findings given by Hussin et al. (2010). On the contrary, Soltan (2002) showed that T. pomace increased the ash content in the carcass; Salama et al. (2015) confirmed that there was no significant difference between T. pomace-based diets and the control diet. Our investigations into the PBM effect on ash content differed from Mahmoud et al. (2023), who ensured that the inclusion of PBM would increase the ash content. Yones and Metwalli (2015) confirmed that the ash content didn’t change among groups. These variations in body composition with other studies could be attributed to several factors. Dietary factors included the high percentage of crude fiber and ash in either azolla or T.pomace. The other factors involved environmental factors like season, temperature and sex ratio, etc.

Hematological findings referred to the absence of any detrimental effect of varying the feedstuffs among the experimental groups. Normal levels of RBCs, Hb, MCV, MCH, MCHC, and WBCs count represented a good indicator of fish health and their physiological state (Prabu et al. 2020). Our results agreed with Sathishkumar et al. (2021), who demonstrated that the addition of PBM had no negative effect on hematological parameters in GIFT tilapia. Also, Refaey et al. (2023) showed that azolla didn’t harm fish hematological parameters when used up to 20% of the diet. Abedalhammed et al. (2020) and Hafez (2024) revealed that T. pomace didn’t affect the hematological indices in both common carp and tilapia.

Generally, there was no tremendous increase or decrease in biochemical indices levels that could refer to any damage or pathological alterations in the physiological functions of fish. ALT, AST, and ALP were measured primarily to assess damage in the liver, as well as serum proteins, which were known to reflect liver health and give a real image of nutritional status (Mahamood et al. 2021; Bojarski et al. 2025). Our investigations into replacing FM with PBM showed that hepatic biomarkers weren’t damaged. Our findings agreed with Mahmoud et al. (2023) and Yones and Metwalli (2015) in Nile tilapia and Li and Cho (2025) in rock fish (*Sebastes schlegeli).* On the contrary Tang et al. (2025) and Yu et al. (2023) found that hepatic indices were greatly affected by PBM addition in mandarin fish and coho salmon (*Oncorhynchus kisutch*), respectively. The results also proved the positive impacts of T. pomace on fish health as they revealed that its addition didn’t harm the hepatic biomarkers despite having a minor increase in the level of AST. Hafez (2024) reported that there was no statistical difference after the addition of T. pomace on ALT, AST levels in Nile tilapia. Amirkolaie et al. (2015) reported that there was no difference in the serum protein levels after adding T. pomace in common carp. Our findings revealed that azolla didn’t have a detrimental effect on the liver indices, despite a slight increase in ALP level compared to the control group, with stability of the rest of the indices, including serum proteins. Magouz et al. (2020),Ismail et al. (2022) and (Sallam et al. 2024) agreed with our findings in GIFT tilapia, Nile tilapia, and red tilapia. On the other side, Ahmed et al. (2023) showed that common carp hepatic indices were greatly affected by the addition of azolla, Refaey et al. (2023) concluded that total protein and globulin increased significantly in Nile tilapia, and Abu-Zahra et al. (2025) reported that the ALP level didn’t change after azolla inclusion.

Kidney function tests were performed to assess renal integrity and physiological state. Urea and creatinine levels didn’t vary among groups, except for a slight increase in the level of creatinine in T_p_ and T_t_, however, these findings remained within the normal range. Our investigations about the PBM addition effect on creatinine differed from Yones and Metwalli (2015) and Mahmoud et al. (2023), who showed that there was no significant difference among groups in Nile tilapia. Hafez (2024) revealed that T. pomace addition didn’t affect the creatinine level. Our Findings about azolla addition resembled the data given by and Magouz et al. (2020),Ismail et al. (2022) and Refaey et al. (2023) in GIFT tilapia and Nile tilapia, respectively.

Lipid profile tests confirmed that adding PBM, T. pomace, and azolla didn’t alter the indices. The results of the PBM inclusion impact on the lipid indices resembled the data presented by Sathishkumar et al. (2021) in GIFT tilapia, and Li and Cho (2025) in rock fish. However Yu et al. (2023) and Tang et al. (2025) confirmed the negative effect of PBM on lipid profile results in coho salmon and mandarin fish, respectively. Magouz et al. (2020) agreed with our data about the azolla effect on lipid indices, but in GIFT Tilapia. On the contrary, Ahmed et al. (2023) reported that the addition of azolla negatively affected cholesterol levels in common carp. Hafez (2024) showed that T. pomace didn’t affect either cholesterol or triglyceride levels. On the other hand, Amirkolaie et al. (2015) confirmed that T. pomace increased cholesterol levels.

Natuzyme didn’t have a clear impact on the level of either hematological or biochemical indices in Nile tilapia. We investigated that using different diets didn’t harm the fish’s health or make alterations to the hemato-biochemical indices. These results also resembled the normal range given in most studies of Nile tilapia (Hrubec et al. 2000; Mauel et al. 2007; Bavia et al. 2024).

Glutathione (GSH) is an integral part of the antioxidant system, particularly in the non-enzymatic process, as it aids in converting hydrogen peroxide (H_2_O_2_) into water and promoting the reduction of lipid hydroperoxides. As a result, variations in the quantifiable levels of GSH were found to be a significant biomarker for assessing the condition of the antioxidant defense system (Motamedi-Tehrani et al. 2025). Moreover, MDA served as a biomarker for oxidative stress associated with lipid peroxidation, which was intensified in environments characterized by heightened oxidative stress (Komal et al. 2024). The results showed that T. pomace addition enhanced the antioxidant status and mitigated MDA levels, which is in agreement with Hafez (2024). It could be attributed to the presence of natural compounds, especially lycopene, which were considered to enhance antioxidant levels and decrease oxidative stress biomarker levels (Tahir et al. 2024). The inclusion of PBM and azolla slightly lowered GSH levels and elevated MDA levels without massive alterations after using these feedstuffs. Mahmoud et al. (2023) indicated that MDA levels didn’t change after total replacement by PBM in Nile tilapia. Dawood et al. (2019) also reported the same findings, but using fermented PBM in Nile tilapia, Li and Cho (2025) showed that PBM didn’t affect superoxide dismutase in rock fish. Yu et al. (2023) reported that using PBM negatively affected MDA levels in coho salmon. Tang et al. (2025) indicated that using PBM in mandarin fish lowered MDA levels and elevated glutathione peroxidase. Abu-Zahra et al. (2025) showed that azolla didn’t affect tissue MDA levels in Nile tilapia, and Ismail et al. (2022) reported the same results using fermented azolla. Refaey et al. (2023) indicated that azolla lowered MDA levels in Nile tilapia. Natuzyme had a positive influence on the antioxidant capacity and lowered MDA levels, which is in agreement with Monier (2020) regarding the use of carbohydrases in common carp.

TNF-α, as a pro-inflammatory cytokine, served as a pivotal mediator in the processes of cellular apoptosis, differentiation, as well as the commencement of inflammatory responses and the modulation of immune activity (Aly et al. 2022). IL-10, as an anti-inflammatory cytokine, triggers the inflammatory response by exerting negative feedback on macrophage activation, while also promoting the production of anti-inflammatory cytokines. (Awad et al. 2022). The slight variation between T_c_ and T_p_ in the levels of TNF-α and IL-10 could be attributed to the fact that FM had a higher content of polyunsaturated fatty acids. These compounds had an anti-inflammatory effect, which mitigated the TNF-α. Tang et al. (2025) confirmed that TNF-α gene expression increased due to PMB inclusion in mandarin fish. Chaklader et al. (2023) also noted that PBM diminished the genetic expression of IL-10, but it increased TNF-α gene expression. The results also revealed that T. pomace altered the immunological parameters by lowering the TNF-α and elevating the IL-10, as it contained lycopene that had strong anti-inflammatory potential (Zhou et al. 2024). Hafez (2024) reported that T. pomace didn’t affect the level of IL-12 gene expression. Azolla didn’t alter the level of cytokines. Natuzyme had a positive influence on the levels of cytokines, which is in agreement with Tao et al. (2025) who used carbohydrases in GIFT Tilapia.

Morphometric analysis of villi height and width, as well as the intestinal absorptive area, could aid in forecasting the mechanism of digestion and absorption in the fish gastrointestinal tract **(**Rašković et al. 2011). The anterior intestinal morphometry revealed that villi length was significantly higher in the control group, followed by the other experimental groups. The absorption surface area was higher in the control group, followed by T_p_ and T_ae_. This means an enhancement in nutrient uptake by providing more contact between digested feed and the intestinal lining through longer villi and more mucosal folds in these groups. So, it improves protein, lipid, and mineral, and vitamin absorption, feed efficiency, strengthens enzyme activity, and supports the intestinal barrier for disease resistance.

Our data about the PBM effect on the intestinal morphometry were similar to Eissa et al. (2022) in Nile tilapia, Chaklader et al. (2023) in barramundi fish (*Lates calcarifer*) and Tang et al. (2025) in mandarin fish. However, Hasan et al. (2024) and Rimoldi et al. (2024) confirmed that PBM didn’t affect the villi parameters in rainbow trout and European seabass *(Dicentrarchus labrax).* Dawood et al. (2020) confirmed that using yeast-fermented PBM in common carp improves the villi height compared to the control group. Our findings about the effect of azolla on the intestinal indices were similar to Refaey et al. (2023) in Nile tilapia. On the contrary Magouz et al. (2020) reported that azolla didn’t affect the villi parameters in GIFT tilapia. Abu-Zahra et al. (2025) showed that azolla improved the villi indices and Ismail et al. (2022) using Bacillus fermented azolla in Nile tilapia. Natuzyme increased the villi parameters numerically in agreement with Tao et al. (2025), who used carbohydrases in GIFT tilapia. However, Hafez et al. (2024) reported that using exogenous enzymes markedly increased the length of the villi when added to T. pomace in Nile tilapia.

Our specimens revealed normal hepato-pancreatic histological structure among the different groups. Mahmoud et al. (2023) reported that there was a mild vacuolation without morphological or pathological alterations after the full replacement of FM with PBM in Nile tilapia. Hasan et al. (2024) and Rimoldi et al. (2024) noted similar observations in rainbow trout and European seabass. On the contrary, Psofakis et al. (2021) showed that PBM made severe alterations to the gilthead seabream and even caused hepatic degeneration. Abu-Zahra et al. (2025) reported that azolla didn’t make any pathological alterations in Nile tilapia.

The kidney and spleen specimens didn’t show significant alterations, except for some minor morphological alterations, which didn’t damage the integrity of these organs. An increase in melano-macrophage centers (MMCs) in T_t_ represented some of these observations, in addition to the high density of lymphoid elements in the white pulp of T_c_ and T_p_. Unlike our investigations, Eissa et al. (2022) noted that MMCs were more diffused in the FM group compared to poultry offal silage.

NF-κB is a significant pro-inflammatory transcription factor that can be triggered by extracellular signals (Baeuerle and Henkel 1994). The immunohistochemical detection of NF-κB expression in the hepatic tissue revealed that photomicrographs showed negative expression among the experimental groups. The statistical analysis of NF-κB expression areas revealed no significant difference among the groups. Tang et al. (2025) confirmed our investigations and showed that PBM didn’t increase the genetic expression of NF-κB in mandarin fish. However, Tao (2025) showed that exogenous enzymes decreased the level of its genetic expression. The inflammation reaction occurred due to internal or external stimuli, which activated NF-κB as a pro-inflammatory mediator. Most studies indicate that inflammatory responses adversely affect muscle protein deposition and growth, suggesting a possible trade-off between immunological function and development in fish (Pooley et al. 2013). Our observations confirmed that the alternative feedstuffs we used didn’t harm the health of tilapias. So, all energy will be directed toward the productive process rather than inflammatory reactions.

Our data revealed that the PBM group was 45% more cost-efficient than the control group, primarily due to the lower cost of PBM compared to fish meal. T_a_ and T_ae_ groups findings was similar to T_c_ and T_p_, indicating azolla potential as a feasible alternative to replace soybean meal and yellow corn. T. pomace-based diets were less efficient than the other groups. El-Sayed (1998) indicated that using PBM was more efficient than FM in terms of the profit index in tilapia. (Fontinha et al. 2021) reported that using higher percentages of PBM instead of FM improved the economic efficiency ratio in gilthead seabream(*Sparus aurata*). Sevgİlİ and Ertürk (2004) reported that PBM was 16% more cost-efficient than the control group with a 20% inclusion level in rainbow trout. On the contrary, Hasan et al. (2024) indicated that using PBM in rainbow trout would give a lesser economic benefit compared to the control. Li and Cho (2025) showed that its use at higher inclusion levels resulted in reduced economic efficiency in rock fish Abou et al. (2007) and Refaey et al. (2023) stated that using azolla at a 20% inclusion level gave the best economic performance. Soltan (2002) and Hussin et al. (2010) indicated that incorporating tomato pomace at a 26% inclusion level was cost-effective in Nile tilapia. This disagreement with our findings may be due to the composition of ingredients, experimental conditions, and environmental conditions, including temperature, etc.

## Conclusion

We concluded that PBM is a low-cost alternative to FM. It compensated for the growth indices investigated and demonstrated that PBM outperformed FM in terms of economic efficiency. The growing performance and economic assessment of Azolla were comparable to those of PBM. While T. pomace exhibited the least favorable findings, presumably due to its elevated fiber content, it can be enhanced through increased enzyme supplementation or advanced processing techniques. Using different alternative feedstuffs didn’t change the dressing percentage or visceral index, but it reduced the hepatosomatic index in the azolla groups. Carcass fat and protein of the azolla group fish were the highest, and in the PBM group, the lowest. Overall physiological findings, including (Hematological, biochemical, redox status, cytokines and histopathological findings) revealed that PBM, T. pomace and azolla didn’t harm the fish health. Natuzyme didn’t affect the growth parameters but positively impacted on the redox status and liver cytokine levels and could potentially make a greater difference by using a higher dose. These alternative feed ingredients offer a sustainable approach to aquaculture by providing locally available, cost-effective resources while minimizing environmental impact through the utilization of agricultural and industrial by-products.

## Data Availability

The datasets generated and/or analyzed during the current study are available from the corresponding author on reasonable request.
